# Link Prediction in Dynamic Social Networks Combining Entropy, Causality, and a Graph Convolutional Network Model

**DOI:** 10.3390/e26060477

**Published:** 2024-05-30

**Authors:** Xiaoli Huang, Jingyu Li, Yumiao Yuan

**Affiliations:** School of Electrical Engineering and Electronic Information, Xihua University, Chengdu 610000, China; jingyuli@stu.xhu.edu.cn (J.L.); yuanyumiao@stu.xhu.edu.cn (Y.Y.)

**Keywords:** link prediction, dynamic social networks, temporal information entropy (TIE), node2vec, causality analysis, graph convolutional networks (GCNs)

## Abstract

Link prediction is recognized as a crucial means to analyze dynamic social networks, revealing the principles of social relationship evolution. However, the complex topology and temporal evolution characteristics of dynamic social networks pose significant research challenges. This study introduces an innovative fusion framework that incorporates entropy, causality, and a GCN model, focusing specifically on link prediction in dynamic social networks. Firstly, the framework preprocesses the raw data, extracting and recording timestamp information between interactions. It then introduces the concept of “Temporal Information Entropy (TIE)”, integrating it into the Node2Vec algorithm’s random walk to generate initial feature vectors for nodes in the graph. A causality analysis model is subsequently applied for secondary processing of the generated feature vectors. Following this, an equal dataset is constructed by adjusting the ratio of positive and negative samples. Lastly, a dedicated GCN model is used for model training. Through extensive experimentation in multiple real social networks, the framework proposed in this study demonstrated a better performance than other methods in key evaluation indicators such as precision, recall, F1 score, and accuracy. This study provides a fresh perspective for understanding and predicting link dynamics in social networks and has significant practical value.

## 1. Introduction

The rapid development of communication, information dissemination and global social interaction has made social networks an integral part of our daily lives [1]. A social network comprises a complex network of connections between nodes, typically visualized as a graph, where nodes represent users or entities and edges describe the relationship or interaction between them. For example, in the network composed of data collected by the Facebook platform [2], nodes represent users registered with platform IDs and edges indicate that there is a friend relationship between User *A* and User *B*. In the network composed of data collected by the DBLP platform [3], nodes represent authors of published articles and edges indicate that Author *A* and Author *B* have jointly published at least one article. In the network composed of data collected by the Amazon platform [3], nodes represent goods and edges indicate that Good *A* and Good *B* are often purchased at the same time. Analyzing the structure and dynamics of social networks is crucial, prompting scholars to explore various applications and conduct research to mine valuable information that facilitates daily life [4], which can be applied to diverse fields [5], including visualization, node classification, system recommendation and link prediction.

**Figure 1:**
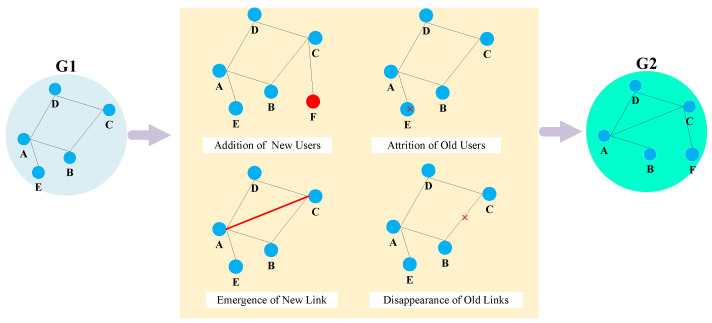
Dynamic social network example.

**Figure 2:**
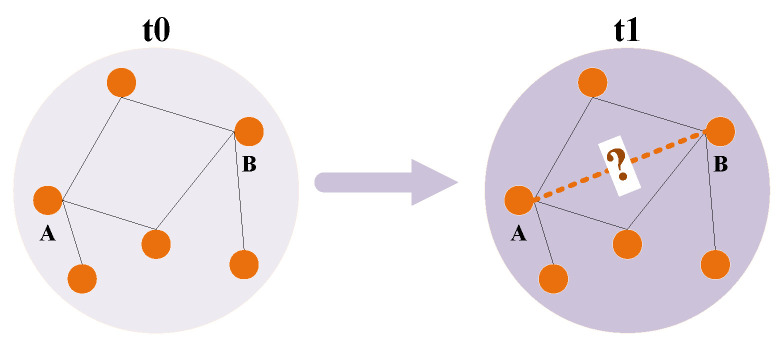
Hypothetical example of dynamic social network link prediction.

In a dynamic social network, new connections continuously emerge, while existing connections disappear or weaken over time, which constitutes a so-called dynamic social network [6]. This dynamic change is a fundamental characteristic of social networks, driven by factors such as the addition of new users, the reduction in existing users, interactions between diverse users and interruptions in connections [7]. This is shown in Figure 1 below. Given this reason, numerous scholars have studied issues related to these connections, and link prediction plays a pivotal role in social network analysis [8]. Link prediction involves forecasting the probability of future connections or relationships between nodes, leveraging existing network topologies and other pertinent attributes [9]. In essence, it aims to identify potential connections within the network that have not yet been established but may form in the future. Similarly, in a small network where Node *A* and Node *B* are not connected at time t0, link prediction predicts whether Node *A* and Node *B* will be connected at time t1, as shown in Figure 2 below.

Link prediction, used to predict the likelihood of future connections between nodes in a dynamic network, remains a vibrant research area. Thus, researchers have studied numerous methods to solve this prediction challenge [10]. Historically, much of the work involves transforming original datasets into time snapshots and subsequently performing prediction tasks, ranging from node similarity and features to deep learning and hybrid methods [11]. Among the fundamental similarity methods is the common neighbor (CN) approach, which predicts links based on the common neighbors between nodes. The Jaccard coefficient quantifies the node similarity by comparing the ratio of common neighbors to total neighbors, and the Adamic–Adar (AA) index assigns a higher importance to common neighbors with fewer connections [12]. Other similarity metrics incorporate global information, considering changes in path lengths between nodes [13], or utilize random-walk-based methods, employing cosine similarity scores to gauge the connection likelihood. While these similarity-based methods are straightforward, they might not fully capture the diverse features influencing node connections in real networks. Therefore, some researchers have also conducted research to explore node features, considering attributes beyond the node structure. Zhang et al. [14] proposed a link prediction method based on non-negative matrix decomposition technology, which reconstructs the correlation between different types of matrices by projecting from the high-dimensional vector space to the low-dimensional vector space, and fused node correlation attributes to extract potential features. Chen et al. [15] proposed a new link prediction model based on deep non-negative matrix decomposition, which utilizes the observed link information of each hidden layer to obtain additional node feature information. In addition, graph embedding, which converts an attribute graph into a vector or a vector set, is an important tool for network mining and can also be used to extract network node features. Perozzi et al. [16] proposed the Deepwalk algorithm and introduced the idea of word embedding in graph embedding, which is equivalent to decomposing the initial matrix into two matrices and then concatenating them to obtain node feature vectors. Tang et al. [17] proposed the LINE algorithm, which is characterized by node vectors derived from two similarity indices. Grover et al. [18] proposed an improved Node2Vec algorithm based on the Deepwalk algorithm, which replaced the random walk strategy with depth-first search (DFS) and breadth-first search (BFS) so as to represent node embeddedness more effectively. Although using node feature engineering has advantages in simplicity and interpretability, its prediction accuracy is lower when used alone. Therefore, some researchers combine feature engineering with deep learning models to improve predictive performance while retaining interpretability. Yi et al. [19] proposed link prediction based on feature extraction combined with a Deep Autoencoder model by learning node features from the adjacency matrix in a hybrid autoencoder reconstruction network. Kumar et al. [20] explored advances in GCNs for social network link prediction, including various GCN architectures, feature engineering, and evaluation metrics. Recently, some researchers have proposed several hybrid models for link prediction to improve its performance [21]. Jiawei et al. [22] proposed a new graph neural network framework, GraphSAGE++, which introduced causal inference into the GraphSAGE model and used aggregation functions to integrate selected neighbor features into the feature vector generation of target nodes. Tan et al. [23] proposed a counterfactual and factual (CF2) reasoning-based deep neural network interpretation and evaluation method for graph prediction. Kumar et al. [24] used the improved Node2Vec combined with an attention mechanism to generate feature vector representations, and then used a hybrid deep learning model for graph prediction.

These studies provide abundant solutions to the link prediction problem. However, many of these methods are based on time snapshots [25], which ignore the temporal characteristics of node interactions within the network. In these processing methods, a large time span is considered, potentially overlooking multiple rapid interactions between nodes [26]. With technological advancements, analyzing vast datasets has become more affordable, facilitating the recording and processing of the timing of each node interaction in the network. Capturing time information for all interactions offers a more comprehensive understanding of each node’s temporal patterns. By utilizing this time information, we can understand the time complexity and unpredictability of each node’s interactions, which are not provided by time snapshots. Meanwhile, some studies have shown that considering temporal [27] and causal characteristics [23] can enhance the link prediction performance. Another shortcoming in the existing literature on dynamic social network link prediction is the lack of attention to the use of node embeddings to generate feature vectors. Node embeddings can effectively generalize to nodes with similar structures, and the learned features are robust and adaptable to various scenarios [28]. Node embedding and machine learning algorithms complement each other, and when combined, they can effectively enhance the link prediction performance [29].

In this work, we propose a novel fusion framework that combines entropy, causality, and a GCN for link prediction in dynamic social networks. Firstly, the original data are collected and processed, retaining only user IDs, user interactions and interaction times. This focus on relevant information improves the efficiency of the learning process. Secondly, we propose an improved Node2Vec algorithm and assign entropy to each user based on their interaction times. We then use entropy as the weighted factor in the random walk of Node2Vec to generate user feature vectors. This method helps produce output results that consider both the interaction times and network structure. Thirdly, the extracted features undergo further processing using causal analysis. We assign a causal intensity score to each user to weight the initial feature vectors after processing. Fourthly, we use the processed feature vectors to generate datasets, which are then divided into training, validation, and testing sets. The training and validation sets are iteratively trained on the specific GCN model to better predict the likelihood of user-to-user relationships and future connections. Finally, we conduct multiple experiments across different datasets and calculate various evaluation metrics. Concurrently, the model we designed is compared with other models based on machine learning, and the obtained results highlight the high performance of our model for dynamic social network link prediction.

The main contributions of this paper are as follows:

1. We design a novel fusion framework that combines entropy, causality, and a GCN for link prediction in dynamic social networks;
2. We propose the concept of Temporal Information Entropy (TIE), which is used as a weighting factor in the Node2Vev random walk. We then introduce an improved Node2Vec algorithm for feature generation, enabling analysis of dynamic social networks from both temporal and structural perspectives;
3. We construct a causality analysis model and use it to process the generated feature vectors, which helps to weight the influence of current node features based on their causal strength;
4. We use a specific optimizer and dynamic learning rate with the GCN model, enabling better capturing of network characteristics and achieving a higher performance output;
5. We conduct repeated experiments on different datasets, and highlight the performance of the proposed fusion framework compared to other models.

The structure of this paper is as follows: Section 1 describes the research background, the definition of link prediction and work by other scholars. Section 2 describes our proposed fusion framework, including its structure and the implementation of each part. Section 3 describes the experimental settings by describing the datasets, related settings, benchmark models and evaluation metrics. Section 4 discusses effect of the sensitivity to positive and negative sample ratios on the prediction performance. Repeated experiments are designed to verify the superiority of our model compared to other models and the confidence interval is analyzed to evaluate the mathematical rationality of the model. Section 5 summarizes the work in this paper and puts forward future development directions.

## 2. Methods

**Figure 3:**
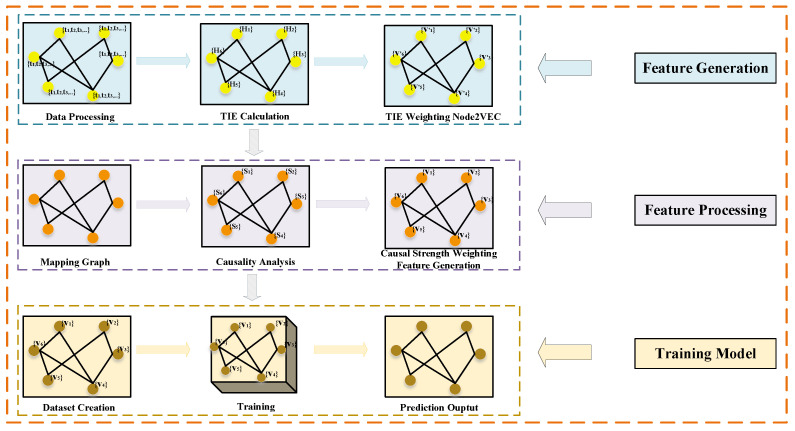
The flowchart of dynamic social network link prediction combining entropy, causality, and GCN models.

This section describes our innovative proposed framework for dynamic social network link prediction. Firstly, we preprocess the collected datasets, focusing on retaining user IDs and interaction timestamps (where the timestamp means the total number of seconds from GMT 1970.01.01 00:00:00 to the deadline), and store them in order, recorded as (u,v,t), indicating that User *u* and User *v* have interacted at time *t*. Secondly, we create a dictionary for each user to store their outgoing and incoming timestamps with other users, denoted as (u:[t0,t1,t2,…]), indicating that User *u* has interacted with other users at t0, t1, t2, etc. Thirdly, we use the above generated dictionary set to calculate a TIE for each user, and then weight them in the Node2Vec algorithm’s random walk to generate the initial features of the network nodes. Fourthly, these initial features are then further processed through a causality analysis to better capture information about important users. Fifthly, the resulting feature representations are then input into GCN models, which are tuned with specific optimizers and learning rates to further improve the prediction performance. The main framework of the model is divided into the following three parts: (i) feature generation based on an improved Node2Vec, (ii) feature processing based on a causality analysis, and (iii) training based on specific GCN models. Figure 3 shows the flowchart of dynamic social network link prediction combining entropy, causality, and GCN models. The models we used for each step are detailed below.

### 2.1. Feature Generation Based on Improved Node2Vec

In this section, we explain the use of the TIE as a weighting factor in random walks based on the Node2Vec algorithm, and then propose the details of feature generation based on the improved Node2Vec algorithm. This section is divided into two parts: (i) Temporal Information Entropy (TIE) and (ii) feature generation of combining Node2Vec and TIE.

#### 2.1.1. Temporal Information Entropy (TIE)

“Entropy” is often used in various fields of science and mathematics, and mostly represents a measure of disorder, uncertainty, and information [30]. For example, entropy in thermodynamics is a measure of the thermal energy of not doing work in a system and Shannon Entropy in information theory is a measure of the uncertainty or information computing in a probability distribution. In this paper, the concept of Temporal Information Entropy (TIE) is applied to dynamic networks containing node, edge and timestamp information, and is a quantitative measure of irregularity or unpredictability over time. A high entropy indicates greater variability in the node interaction time, while a low entropy indicates more regularity in the node interaction time.

Using the preprocessed data obtained in the previous step, all incoming and outgoing timestamps of each user and other users are stored in dictionary form (nodes represent users), and the sorted calculation process is as follows:

1. Sequentially record the timestamp difference value for each node between time *i* and time i+1 and denote it as $\Delta t_{i}$;
2. Sequentially sum the timestamp difference values for each node and denote it as Δt;
3. Sequentially calculate the probability of each node’s timestamp difference value, as shown in Equation (1) below. Given the low probability of interactions transpiring at identical time intervals in the real world, each node’s timestamp difference signifies a unique scenario. The proportion of each timestamp difference to the sum of all timestamp differences is used as a probability for subsequent operations, where $p_{i}$ represents the probability of each node’s timestamp difference occurring, $\Delta t_{i}$ represents each node’s timestamp difference value between time *i* and time i+1, and Δt represents the sum of timestamp differences for each node:
  (1) $$p_{i}=\frac{\Delta t_{i}}{\Delta t},$$
4. Sequentially compute the Shannon Entropy by using the above probabilities and then sum them them up. Next, the initial value of time information entropy of each node can be obtained, as shown in Equation (2) below, where $H_{TIE_{0}}$ represents the initial non-normalized Temporal Information Entropy:
  (2) $$H_{TIE_{0}}=\sum p_{i}\times log_{2}p_{i},$$
5. Sequentially standardize the initial entropy of each node, and the final output TIE is obtained as shown in Equation (3) below, where $H_{TIE}$ represents each node’s TIE and *N* represents the total number of nodes in the current network:
  (3) $$H_{TIE}=\frac{H_{TIE_{0}}}{\sum_{N}H_{TIE_{0}}}\times N,$$

By using the probability derived from the timestamp differences and combining it with the Shannon Entropy to capture the information about node interaction time, we provide a standardized and interpretable calculation method for calculating the TIE. This method helps identify differences in the irregularity of node interaction time changes in dynamic networks and promotes the analysis and understanding of time dynamics in various fields.

#### 2.1.2. Feature Generation of Combining Node2Vec and TIE

In order to obtain feature representations of users, we first collect the data and then use graph topology along with other relevant information to generate them. Thus, we modify the existing Node2Vec diagram embedding method. The feature vectors are generated by nodes that represent information about the user in a low-dimensional feature space of dimension *d* (the length of feature vectors we set), which helps to capture details about other users associated with the user.

**Figure 4:**
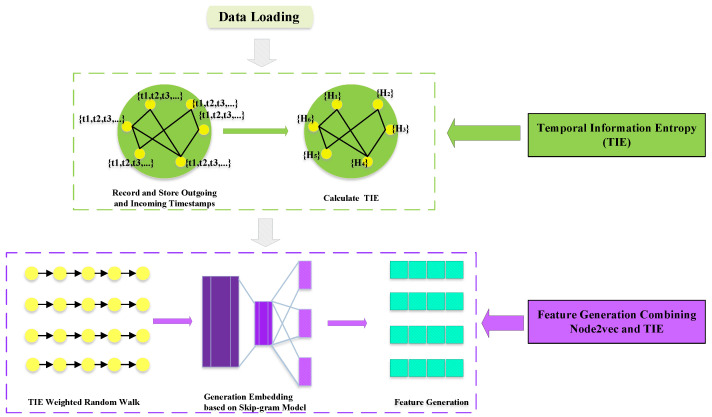
The flowchart of generated features combining the TIE and Node2Vec.

In our proposed improved Node2Vec feature generation method, we first perform random walks on the graph to capture local and global structure information and then use traversal to generate features for each node. Next, we apply the Skip-gram model to these features to generate node feature vectors. At the core of algorithm is a random walk, which explores the network by traversing the graph in a series of steps. At each step, the random walk moves from the current node to the adjacent node according to the probability distribution of the balanced BFS and DFS strategies. The random walk is controlled by two deviation parameters: the return parameter (*p*) and the input/output parameter (*q*). Here, *p* specifies the probability of revisiting the current node and *q* determines the probability of visiting a node far from the current node. For example, considering the random walk from Node $v_{i}$ to Node $v_{j}$, the next in the process is Node $v_{k}$. The calculation of this transition probability requires the aforementioned search bias, as shown in Equation (4) below:

(4) $$\alpha_{pq}\left(v_{i},v_{k}\right)=\left\{\begin{array}{c} \frac{1}{p},d_{v_{i}v_{k}}=0\\ 1,d_{v_{i}v_{k}}=1\\ \frac{1}{q},d_{v_{i}v_{k}}=2 \end{array}\right.,$$

where $\alpha_{pq}\left(v_{i},v_{k}\right)$ represents the search deviation in the random walk process and $d_{v_{i}v_{k}}$ represents the shortest path between Node $v_{i}$ and Node $v_{k}$. Then, the above search bias is used to calculate the non-normalized transition probability, as shown in Equation (5) below:

(5) $$\pi_{v_{i}v_{k}}=\alpha_{pq}\left(v_{i},v_{k}\right)\cdot w_{v_{i}v_{k}},$$

where $\pi_{v_{i}v_{k}}$ represents the non-normalized transition probability and $w_{v_{i}v_{k}}$ represents the edge weight of Node $v_{i}$ and Node $v_{k}$ (the default value of unweighted graphs is generally 1). The dynamic social network graph after processing is an unweighted graph, and the edge weight is fixed to 1. Therefore, in the improvement of the original Node2Vec, in order to calculate the non-normalized transition probability, we take the TIE of the next Node $v_{k}$ as the weighting factor of the original search and consider the node structure and time change factors, which is conducive to effective and accurate analyses of dynamic social networks. The improved non-normalized transition probability is shown in Equation (6) below:

(6) $$\pi_{v_{i}v_{k}}=c1\times \alpha_{pq}\left(v_{i},v_{k}\right)+c2\times H_{t}\left(v_{k}\right),$$

where $H_{t}\left(v_{k}\right)$ represents the TIE of $v_{k}$ and c1 and c2, respectively, represent the weight of non-normalized transfer probability considering the structure and time factors. Then, the final transition probability is shown in Equation (7) below.

(7) $$p\left(c_{i}=v_{k}|c_{i-1}=v_{i}\right)==\left\{\begin{array}{c} \frac{\pi_{v_{i}v_{k}}}{\sum \pi_{v_{i}v_{k}}},\left(v_{i},v_{k}\right)\in E\\ 0,\mathrm{other} \end{array}\right.,$$

where *E* represents the set of edges in the network. The sequence of nodes generated by the modified random walk above is then used to train the Skip-gram model in Word2Vec. The model learns node embeddings by predicting context nodes based on a given target node, and can effectively learn high-quality node embeddings from random walk sequences to generate feature vectors for each node. Therefore, under the improved Node2Vec algorithm combined with the entropy proposed by us, an n×d-dimensional eigenmatrix can be obtained, where *n* represents the number of nodes (i.e., users) in a dynamic social network. Figure 4 below shows the flowchart of our proposed improved Node2Vec feature generation method. The generated feature vectors will be further processed and used in the following steps.

### 2.2. Feature Processing Based on a Causality Analysis

Further processing of the feature vectors generated in the previous step can more effectively capture the relationship between nodes in the network. Thus, we construct a causal analysis model for social networks, the output of which assigns a causal strength score to each node in the network. In a social network, a node with a strong causal strength indicates that the corresponding user has a significant influence over other users in the network, possibly serving as the center or focus. These nodes may play a key role in facilitating communication, information dissemination, or collaboration among other network members. Incorporating causal strength weighting into existing feature vectors can more accurately predict future connections. The specific experimental steps are as follows:

1. Draw a network map based on the data;
2. Analyze node correlation. Firstly, introduce the concept of mixed centrality (mixing degree centrality, closeness centrality and eigenvector centrality). Secondly, set a threshold, and select nodes larger than the threshold as the current nodes for correlation;
3. Set up counterfactual experiments for a causality analysis. Firstly, estimate the node selected in step 2 as the “cause”. Secondly, carry out a path analysis between this current node and other nodes, referred to as target nodes, within the network, examining these paths sequentially. If more paths contain target nodes, this indicates that deleting the current node will cause changes in the target node, confirming the current node as the “cause” of the target node. Otherwise, there is no causal relationship between the current node and the target node. Thirdly, count the number of times the current node is used as a “cause”, denoted as causal strength $S_{v}$;
4. Standardize the causal strength obtained from the casual analysis and then incorporate it into the existing feature vector generation method, as shown in Equations (8) and (9) below, where $\bar{s_{v_{i}}}$ represents the standardized causal strength value, $v_{0}$ represents the feature vectors generated based on the improved Node2Vec, *v* represents feature vector processing based on the causal analysis, and *c* represents a constant (default 0.1, considering the case where the causal intensity is 0).
  (8) $$\bar{s_{v_{i}}}=\frac{s_{v_{i}}}{\sum_{j}^{n}s_{v_{j}}}\times n,$$
  (9) $$v=\left(\bar{s_{v_{i}}}+c\right)\times v_{0},$$

**Figure 5:**
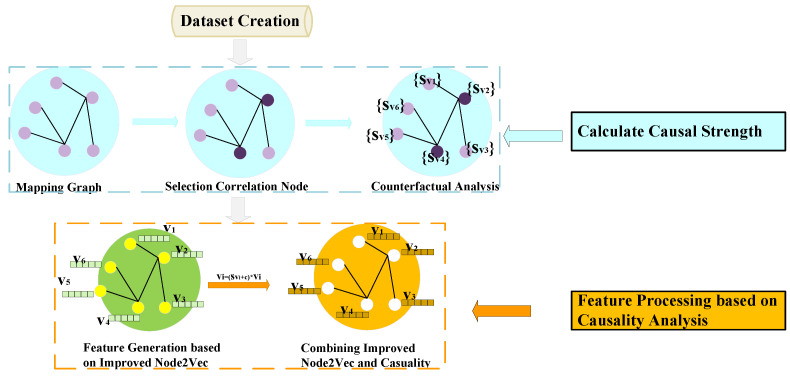
The flowchart of feature vector processing based on a causality analysis.

Feature vector processing based on a causality analysis can better reflect the causality relationships between nodes. This ensures that the generated feature vectors capture not only the structural properties of the network, but also the causal relationships between nodes, providing more informative features for predicting future connections. Figure 5 below shows the flowchart of feature vector processing based on a causality analysis.

### 2.3. Training Based on Specific GCN Models

This section describes some work we have undertaken for training with specific GCN models. Primarily, we need to process the previously processed data into a form that includes the node feature matrix, edge link information and labels. However, in this case, an edge consists entirely of positive samples, and its label is uniformly set to 1 (as the original dataset comprises node pairs with edges). Using only positive samples for model training will make the model less generalizable to new data, leading to a poor performance. Therefore, we need to add negative samples to the original data, representing edges that do not exist in the current network, with their labels set to 0. Given that the focus is on predicting future connections between nodes, the number of negative samples may be smaller than the number of positive samples. By considering both positive and negative samples during training, the model can make more robust and reliable predictions. After this processing, the dataset is divided into a training set, a verification set and a testing set according to a specified proportion, and the training set and verification set are then combined to train the model. The model’s link prediction performance is evaluated on the testing set.

**Figure 6:**
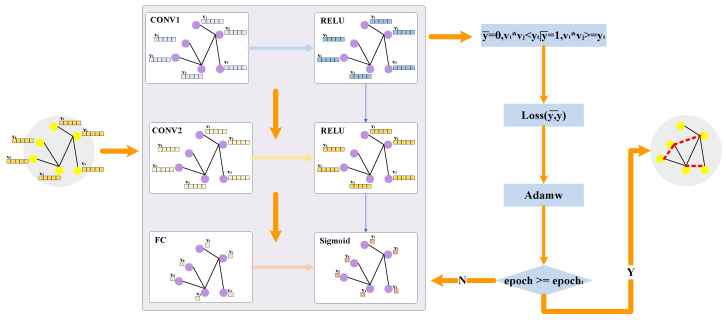
The flowchart based on specific GCN model training.

After successfully creating a balanced dataset, we begin training the deep learning model. In this study, we have tested GraphSAGE, Deep Autoencoder and GCN models. Finally, we used the Adamw optimizer and a dynamic learning rate with the GCN model for the final link prediction. Figure 6 below shows the flowchart based on the specific GCN model training, where $\bar{y}$ represents the predicted output of a specific GCN model, $y_{t}$ represents the threshold for judging edge connections (default 0.5), and $epoch_{t}$ represents the number of experiment iterations.

In addition, we use feature generation based on the improved Node2Vec and feature processing based on a causal analysis as inputs for node feature vectors for the specific GCN model, introducing temporal and causal features of node interactions into our model to help make more accurate predictions.

## 3. Experiments

This section describes our procedures for predicting link changes in dynamic social networks, including the datasets, experimental settings, baseline models and evaluation metrics.

### 3.1. Dataset Introduction

We use three small-scale, real, complex network datasets as research objects. The Email and CollegeMsg datasets are based on timestamped edge network datasets from Stanford University’s large network dataset collection (download link: https://snap.stanford.edu/data/#temporal, accessed on 10 April 2024). The Hypertext dataset is from SocioPatterns, an interdisciplinary research collaboration (download link: http://www.sociopatterns.org/datasets/, accessed on 10 April 2024). These three datasets are described below. In addition, Table 1 shows the node numbers, edge numbers and time information of these three datasets.

The Email network dataset [31] is made up of email communications from a large European research institution, including all incoming and outgoing emails between its members. (u,v,t) means that Member *u* sent an email to Member *v* at time *t*.

The CollegeMsg social network dataset [32] is made up of private messages sent by universities in California on online networks. On this social network, users can search and contact others based on their profile and then initiate conversations. (u,v,t) indicates that User *u* sent a private message to User *v* at time *t*.

The Hypertext conference network dataset [33] was collected during the ACM HyperText2009 conference. Participants voluntarily wore radio badges to observe their face-to-face contact information. In addition, the time information for this dataset was recorded at intervals and it requires timestamp conversion. (u,v,t) indicates that Participant *u* and Participant *v* had face-to-face contact at time *t*.

### 3.2. Experimental Settings

The experiment was conducted on a 64 bit Windows 11 operating system utilizing an Intel Core i7 processor. The programming language of choice was Python3.8, and PyCharm2020 was used as the code execution software. The deep learning framework implemented was Torch. In order to evaluate the efficacy of our proposed hybrid model, which integrates entropy, causality and a GCN model for dynamic social network link prediction, we have selected various other hybrid models for comparison. These include models that couple features with Deep Autoencoder, features with GraphSAGE, and features with a GCN. The processed dataset was subdivided into training, validation, and testing sets in the proportion of 8:1:1, with the experiment being repeated multiple times. Furthermore, during the training process, the model’s maximum number of iterations was capped at 200.

### 3.3. Benchmark Models

The following is an introduction to the model we use in experiments.

1. Deep Autoencoder: Deep Autoencoder is an artificial neural network model used for unsupervised learning and dimensionality reductions. It consists of an encoder network that maps input data to a low-dimensional latent space and a decoder network that reconstructs input data from a latent space representation, where *z* represents the first part of the composition, as shown in Equation (10) below. $\hat{x}$ represents the second part of the composition, as shown in Equation (11) below. The Deep Autoencoder link prediction process after training is shown in Equation (12):
  (10) $$z=f(W_{e}x+b_{e}),$$
  (11) $$\hat{x}=g\left(W_{d}z+b_{d}\right),$$
  (12) $$DeepAutoencoder=DeepAutoencoderPrediction(D_{train}),$$
2. GraphSAGE (Graph Sample and Aggregate): GraphSAGE is a graph neural network architecture that captures graph structure information by sampling and aggregating information from each node’s domain, where N(v) represents the neighbor of Node *v*. $h_{v}^{\left(l+1\right)}$ represents the output of Node *v* at layer l+1, as shown in Equation (13) below. The GraphSAGE link prediction process after training is shown in Equation (14):
  (13) $$h_{v}^{\left(l+1\right)}=\sigma \left(\phi \left(\left\{h_{u}^{\left(l\right)}:u\in N\left(v\right)\right\}\right)W^{\left(l\right)}\right),$$
  (14) $$GraphSAGE=GraphSAGEPrediction(D_{train}),$$
3. Graph Convolutional Networks (GCNs): GCNs are a graph neural network designed for manipulating graph-structured data, learning by aggregating information from adjacent nodes, where $h_{v}^{\left(l+1\right)}$ represents the output of Node *v* at layer l+1, as shown in Equation (15) below. The GCN link prediction process after training is shown in Equation (16) below:
  (15) $$h_{v}^{\left(l+1\right)}=\sigma \left(\sum_{u\in N(v)}\frac{1}{c_{u,v}}W^{\left(l\right)}h_{u}^{\left(l\right)}\right),$$
  (16) $$GCN=GCNPrediction(D_{train}),$$

### 3.4. Evaluation Metrics

We use precision, recall, the F1 score, and accuracy to evaluate the performance of our proposed dynamic social network link prediction method combined with entropy, causality and GCN models. These metrics are widely used to evaluate the performance of link prediction and have the ability to accurately analyze whether the model correctly predicts connections between nodes in the network.

Precision refers to the proportion of the number of correctly predicted positive samples to the number of predicted positive samples. In link prediction, it can be expressed as the proportion of the number of correctly predicted node–pair connections to the number of all predicted connections, as shown in Equation (17) below:

(17) $$Precision=\frac{TP}{TP+FP},$$

Recall refers to the proportion of the number of correctly predicted positive samples to the number of actual positive samples. In link prediction, it can be expressed as the proportion of the number of correctly predicted node–pair connections to the number of actual connections, as shown in Equation (18) below:

(18) $$Recall=\frac{TP}{TP+FN},$$

The F1 score is the harmonic average of accuracy and recall, providing a balanced measure of a model’s performance. It considers both the precision and recall, making it suitable for situations with an imbalance between positive and negative instances. It is shown in Equation (19) below:

(19) $$F1=\frac{2\times Precision\times Recall}{Precisoin+Recall},$$

Accuracy refers to the proportion of the number of correctly predicted samples to the number of total samples, as shown in Equation (20) below:

(20) $$Accuracy=\frac{TP+TN}{TP+TN+FP+FN},$$

where TP (True Positive) indicates correctly predicted positive samples, meaning the predicted positive samples are actually positive samples. FP (False Positive) indicates incorrectly predicted positive samples, meaning the predicted positive sample is actually negative. TN (True Negative) indicates a correctly predicted negative sample, meaning the predicted negative sample is actually a negative sample. FN (False Negative) indicates an incorrectly predicted negative sample, meaning the predicted negative samples are actually positive samples. These evaluation metrics provide different perspectives on the model’s performance in the link prediction task: precision focuses on the correctness of the correct connection predictions, recall emphasizes the model’s ability to capture all the correct connections, the F1 score balances precision and recall, and accuracy measures the overall correctness of the model’s predictions.

## 4. Results

To verify the performance of our proposed dynamic social network link prediction method combining entropy, causality, and a GCN model, we compared it with several other hybrid algorithms. Among them, several hybrid algorithms were designed specifically with reference to the work of Khanam et al. [34], who combined Node2Vec with a deep learning model and optimized the model performance by comparing the design methods of different optimizers. In addition, the Node2Vec algorithm we chose in the comparison method is based on the research of Grover et al. [18]; the Deep Autoencoder model is based on the research of Yi et al. [19]; the GraphSage model is based on the research of Hamilton et al. [35]; and the selection of a GCN model is based on the research of Zhang et al. [36]. Specifically, the method using the Node2Vec algorithm for feature extraction and then combining it with a Deep Autoencoder model (Adam optimizer; learning rate of 0.01) for prediction is denoted as ①; the method using the Node2Vec algorithm for feature engineering extraction and then combining it with the DeepAutoencoder model (Adamw optimizer; dynamic learning rate) for prediction is denoted as ②; the method using the Node2Vec algorithm for feature extraction and then combining it with the GraphSAGE model (Adam optimizer; learning rate of 0.01) is denoted as ③; the method using the Node2Vec algorithm for feature extraction and then combining it with the GraphSAGE model (Adamw optimizer; dynamic learning rate) is denoted as ④; the method using the Node2Vec algorithm for feature extraction and then combining it with the GCN model (Adam optimizer; learning rate of 0.01) is denoted as ⑤; and the method using our proposed combination of entropy, causality, and a GCN model is denoted as ⑥. All the average results presented below are based on the average of ten repeated runs.

### 4.1. Sensitivity to the Positive and Negative Sample Ratio

**Figure 7:**
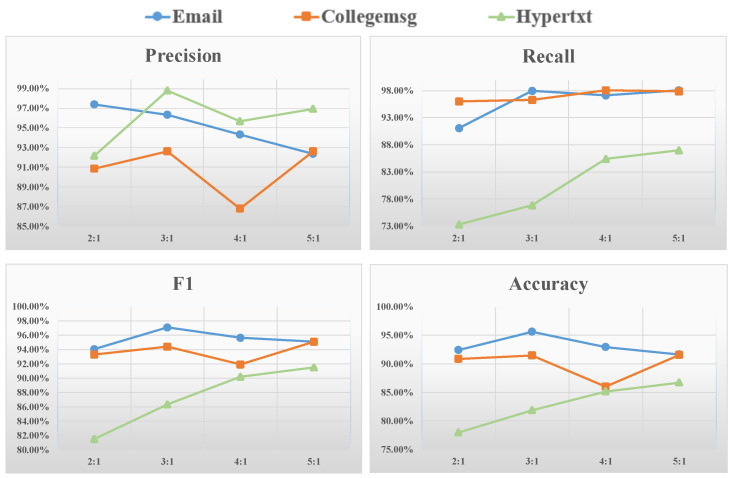
The influence of positive and negative sample proportions on the evaluation metrics obtained by our proposed method on different datasets.

As mentioned in Section 2.3, we considered the proportion of positive and negative samples in the datasets to improve the prediction effect. In this section, we examine the sensitivity of our proposed model to different sample ratios and aim to determine the most suitable ratio. We conducted experiments using four different positive and negative sample ratios: 2:1, 3:1, 4:1 and 5:1. The results using these different sample ratios across all datasets in our proposed method are shown in Figure 7.

As can be seen in Figure 6, in the Email dataset, the precision is highest when the ratio is 2:1, the recall is highest when the ratio is 5:1, F1 is highest when the ratio is 3:1, and the accuracy is highest when the ratio is 3:1; in the CollegeMsg dataset, the precision is highest when the ratio is 3:1, the recall is highest when the ratio is 5:1, F1 is highest when the ratio is 5:1, and the accuracy is highest when the ratio is 5:1; and in the Hypertext dataset, the precision is highest when the ratio is 3:1, the recall is highest when the ratio is 5:1, F1 is highest when the ratio is 5:1, and the accuracy is highest when the ratio is 5:1. Therefore, based on the above performance, it can be seen that when the ratio of positive and negative samples is 5:1 (the negative sample number is 20% of the positive sample number), our proposed method has the best comprehensive performance for each dataset and each evaluation metric. This is because we tend to train using connected samples more during the link prediction process. Therefore, we chose the ratio of positive and negative samples as 5:1, which also provides enough appropriate training samples for our model.

### 4.2. Email Dataset

**Figure 8:**
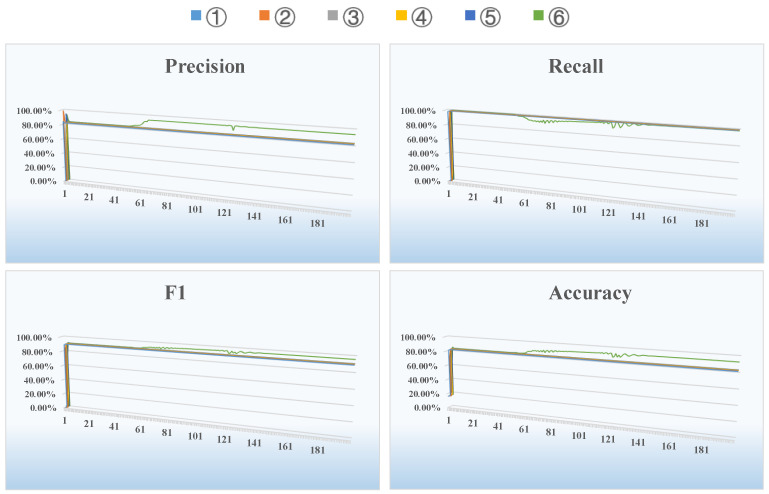
Results of evaluation metrics after 200 iterations of different methods on the Email dataset.

Figure 8 shows the changes in all evaluation metrics during an iteration of a run on the Email dataset using the six methods mentioned earlier. It can be clearly seen from Figure 8 that as the number of iterations increases, the precision, F1 and accuracy of our proposed algorithm are superior to the other five algorithms. By the 60th iteration, the algorithm reaches convergence, and each evaluation metric stabilizes. Table 2 below shows average results of the above six methods for all evaluation metrics after multiple runs of the Email dataset.

From an analysis of Table 2, we can see that for this social network, our proposed method combining entropy, causality and a GCN has the best performance in terms of the precision, F1 and accuracy. Compared to the second best performing methods, our approach shows improvements of 5.95% in precision, 2.79% in the F1 score, and 5.45% in accuracy, achieving better results than other feature engineering plus deep learning models. This shows that our combined approach for improving data extraction feature engineering and using a specific GCN is optimal. The improved results obtained by our method can be attributed to the additional node information captured by the improved feature engineering.

### 4.3. CollegeMsg Dataset

**Figure 9:**
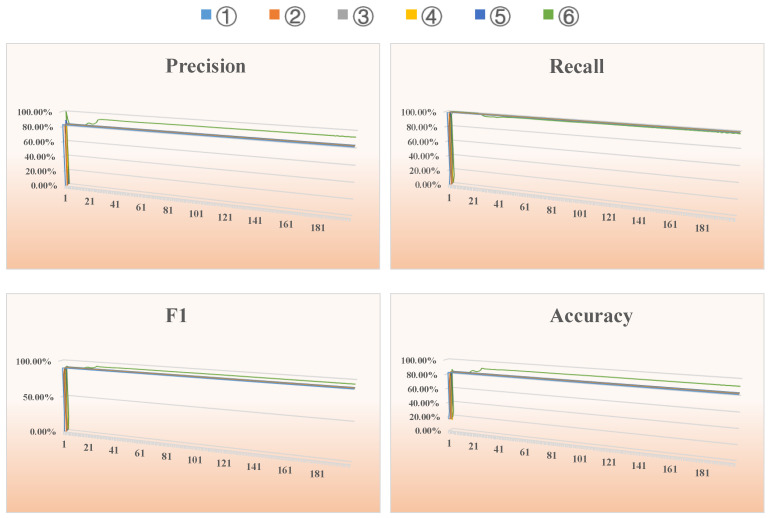
Results of evaluation metrics after 200 iterations of different methods on the CollegeMsg dataset.

Figure 9 shows the changes in all evaluation metrics for California colleges during an iteration of a run on the online social network CollegeMsg dataset using the six methods indicated above. It can clearly be seen from Figure 9 that as the number of iterations increases, the precision, F1 and accuracy of our proposed algorithm are superior to the other five algorithms. By the 40th iteration, the algorithm reaches convergence, and each evaluation metric stabilizes. Table 3 below shows the average results of the above six methods for all evaluation metrics after multiple runs on the online social network CollegeMsg dataset.

From an analysis of Table 3, we can see that for this social network, our proposed method combining entropy, causality and a GCN has the best performance in terms of precision, F1 and accuracy, and the performance increases by 3.97%, 1.39% and 2.95%, respectively, compared to the other state-of-the-art methods. When the optimizer in method ① is changed from Adam to Adamw and the learning rate is changed from 0.01 to a dynamic method ②, the performance of method ③ compared to method ④ and method ⑤ compared to method ⑥ is improved to some extent according to the same logic. This shows that the adjustments and improvements we have made in the GCN model are effective for link prediction.

### 4.4. Hypertext Dataset

**Figure 10:**
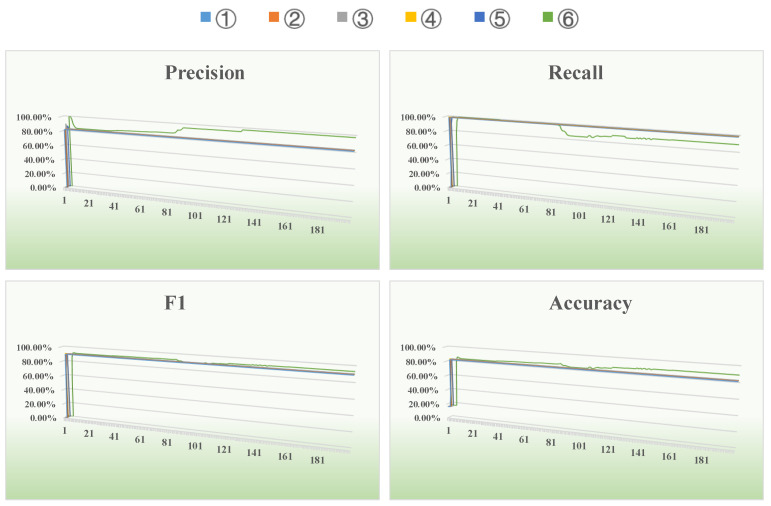
Results of evaluation metrics after 200 iterations of different methods on the Hypertext dataset.

As illustrated in Figure 10, we observe variations in all evaluation metrics throughout one run on the Hypertext dataset of a social network that implements face-to-face interactions using the six methods previously mentioned. From Figure 10, it becomes apparent that as we increase the number of iterations, the precision, F1 score, and accuracy of our proposed algorithm are higher those of the other five algorithms. Despite minor fluctuations in the convergence precision of our proposed algorithm later in the iteration, it essentially stabilises by the 80th cycle. Table 4 provided below presents the average results of the six methods for all evaluation metrics, calculated after carrying out numerous runs on the Hypertext dataset.

Upon evaluating the data in Table 4, it is evident that our proposed method—a fusion of entropy, causality and the GCN model—exhibits the optimal performance in terms of precision, F1 score, and accuracy for this social network. There is a marked improvement of 10.09%, 0.37%, and 2.34%, respectively, when compared to the next best methods. When set against other hybrid methods, ours undoubtedly achieves the best results across these three metrics. This suggests that our improved Node2Vec method, which incorporates Temporal Information Entropy (TIE) and applies a causality analysis to obtain node embedding representations, can extract a more detailed set of node feature information. Complementing this, the training carried out based on particular GCN models allows us to secure better results. This thus substantiates the effectiveness of our fusion framework that combines entropy, causality, and the GCN model for predicting links in social networks.

### 4.5. Confidence Interval Analysis

Table 5 shows the confidence intervals for accuracy based on all datasets. Accuracy is a key index used to evaluate the overall performance in model prediction. The confidence interval represents the likelihood that the predicted true value will fall near the measured result, providing a measure of confidence about the observed value. If the value we find falls within this range, we can say that our value is accurate. For the accuracy we observed, we used 2000 accuracy values obtained from 200 iterations and 10 repetitions of proportional datasets as the total sample. A total of 200 values were randomly selected as samples of the confidence interval, and then the confidence level and distribution were set by finding the mean and standard deviation of the sample. Then, we obtained the confidence interval for the population average. With the same sample size, a higher confidence level leads to a wider confidence interval. For example, the 95% confidence interval of the CollegeMsg dataset is [90.50,91.48] and the 99% confidence interval is [90.35,91.64]. This indicates that if we run our proposed method again for social network link prediction, 95% of the predicted accuracy values will be within the range of [90.50,91.48], and 99% of the predicted accuracy values will be within the range of [90.35,91.64].

A comparative analysis of the data in Table 2, Table 3, Table 4 and Table 5 provides further validation of our method’s performance. For the Email dataset, the accuracy is registered at 91.61, which approaches the upper 99% confidence interval. Notably, even at its lower 99% confidence limit of 88.68, this figure still surpasses the results from the other five methods. Moving on to the CollegeMsg dataset, the accuracy stands at 91.53, falling comfortably within its 99% confidence interval. The lower limit of this interval, at 90.35, proves superior when compared to the corresponding limits of the other five methods. Lastly, the Hypertext dataset offers an accuracy of 86.00, which is close to its upper 99% confidence interval. Moreover, its lower confidence limit of 84.93 outperforms those of the other five methods. Collectively, these results indicate that the superior performance of our proposed method is not only statistically robust but also intrinsically proven. This analysis provides solid evidence of the mathematical soundness and effectiveness of our proposed method.

### 4.6. Complexity Analysis

The proposed fusion framework combining entropy, causality and a GCN model is composed of three parts: feature generation based on an improved Node2Vec, feature processing based on a causality analysis and training based on specific GCN models. The time complexity of these three parts is briefly introduced in the following:

1. Feature generation based on improved Node2Vec: The main time consumption of this part is linked to the Skip-gram model’s iterative training to generate features, and the complexity is roughly $O(E\cdot L\cdot D\cdot W\cdot epoch_{Skip-gram})$, where *E* represents the edge number, *L* represents the random walk length, *D* represents the eigenvector dimension, *W* represents the random walk numbers, and $epoch_{Skip-gram}$ represents the Skip-gram model iteration number;
2. Feature processing based on a causality analysis: The main time consumption of this part is to determine whether there is causality between nodes based on the path, and the complexity is roughly $O(N^{2}\cdot path\cdot pathlength)$, where *N* represents nodes number, path represents paths number, and pathlength represents path length;
3. Training based on specific GCN models: The main time consumption of this part is linked to the GCN model’s iterative training for link prediction, and the complexity is roughly $O(D\cdot E\cdot layers\cdot epoch_{GCN}\cdot K)$, where *D* represents the eigenvector dimension, *E* represents the edge number, layers represents the convolution layer number, $epoch_{GCN}$ represents the GCN model iteration number, and *K* represents the repeated experiment number.

According to the above content, the time complexity of this fusion framework is roughly $O(E\cdot L\cdot D\cdot W\cdot epoch_{Skip-gram}+N^{2}\cdot path\cdot pathlength+D\cdot E\cdot layers\cdot epoch_{GCN}\cdot K)$. Its time complexity is related to the number of nodes, the number of edges, the length of random walks, the number of random walks, the dimension of the eigenvectors, the number of iterations of the Skip-gram model, the number of paths, the length of paths, the number of iterations of the GCN model, and the number of experiments. Thus, the larger the network size, the higher the time complexity and the longer the algorithm runtime.

## 5. Conclusions

Link prediction, widely utilized in sociology, computer science, and other associated fields, aims to forecast the pairings of nodes likely to form future connections. When integrated into dynamic social networks, link prediction can suggest new associations for network users, thereby amplifying user engagement and stimulating the growth of social networks. In recent years, a substantial number of researchers have shifted their focus to link prediction in the realm of dynamic social networks. In this study, we propose a novel methodology that amalgamates entropy, causality, and a GCN model to address the issue of link prediction in dynamic social networks. The first step involves preprocessing the original data to extract and record the timestamp information of node interactions. Following this, we introduced the concept of Temporal Information Entropy (TIE) and incorporated it into the Node2Vec algorithm’s random walk component. This addition generates initial feature vector representations for nodes in the graph, taking into account both temporal and structural influences. Subsequently, we constructed a causality analysis model to carry out secondary processing on the previously generated feature vectors, significantly improving the features and highlighting the impact of crucial nodes. We then tailored the proportion of positive and negative samples within the datasets, with a particular focus on the training process of positive samples. Ultimately, a specific GCN model was used to carry out the training. The methodology was put to the test on several real-world social networks, examining the precision, recall, F1 score, and accuracy metrics. The results indicate that our proposed method showcases an unparalleled comprehensive performance, backed by mathematical logic. Although our proposed method outperforms others in terms of performance, it only considers the user interaction time, neighboring nodes, and network structural properties, leading to feature vectors with restricted node information.

## Figures and Tables

**Table 1:** Real-world network datasets.

Name	Nodes	Edges	Description
Email	986	332,334	Research emails between institutional users.
CollegeMsg	1899	59,835	Research messages on platforms like Facebook.
Hypertext	113	20,818	Research face-to-face contact between participants.

**Table 2:** Average results of different methods (Email dataset).

Model	Precision	Recall	F1	Accuracy
①	83.33	1	90.90	83.33
②	83.30	1	90.89	83.30
③	83.39	1	90.94	83.39
④	83.36	1	90.93	83.36
⑤	86.42	99.25	92.33	86.16
⑥	92.37	98.05	95.12	91.61

**Table 3:** Average results of different methods (CollegeMsg dataset).

Model	Precision	Recall	F1	Accuracy
①	83.35	1	90.92	83.35
②	83.40	1	90.95	83.40
③	83.39	1	90.94	83.39
④	88.16	96.31	91.86	85.68
⑤	88.66	99.72	93.72	88.58
⑥	92.63	97.83	95.11	91.53

**Table 4:** Average results of different methods (Hypertext dataset).

Model	Precision	Recall	F1	Accuracy
①	83.23	1	90.85	83.23
②	83.26	1	90.88	83.26
③	83.43	1	90.97	83.43
④	83.39	1	90.94	83.39
⑤	86.50	96.58	90.76	83.66
⑥	96.59	86.40	91.13	86.00

**Table 5:** Confidence interval for the accuracy of different methods.

Confidence	Email	CollegeMsg	Hypertext
90%	[89.20, 90.99]	[90.58, 91.40]	[85.06, 85.50]
95%	[89.02, 91.16]	[90.50, 91.48]	[85.02, 85.54]
98%	[88.82, 91.36]	[90.41, 91.57]	[84.97, 85.59]
99%	[88.68, 91.50]	[90.35, 91.64]	[84.93, 85.63]

## Data Availability

Data are contained within the article.

## Abbreviations

The following abbreviations are used in this manuscript:

MDPI	Multidisciplinary Digital Publishing Institute
GCN	Graph Convolutional Network
TIE	Temporal Information Entropy
DFS	Depth-First Search
BFS	Breadth-First Search
